# UAV imagery data and machine learning: A driving merger for predictive analysis of qualitative yield in sugarcane

**DOI:** 10.3389/fpls.2023.1114852

**Published:** 2023-01-26

**Authors:** Marcelo Rodrigues Barbosa Júnior, Bruno Rafael de Almeida Moreira, Romário Porto de Oliveira, Luciano Shozo Shiratsuchi, Rouverson Pereira da Silva

**Affiliations:** ^1^ Department of Engineering and Mathematical Sciences, School of Agricultural and Veterinarian Sciences, São Paulo State University (Unesp), São Paulo, Brazil; ^2^ AgCenter, School of Plant, Environmental and Soil Sciences, Louisiana State University, Baton Rouge, LA, United States

**Keywords:** remote sensing, brix, sucrose, ripening, *Saccharum* spp., smart harvest

## Abstract

Predicting sugarcane yield by quality allows stakeholders from research centers to industries to decide on the precise time and place to harvest a product on the field; hence, it can streamline workflow while leveling up the cost-effectiveness of full-scale production. °Brix and Purity can offer significant and reliable indicators of high-quality raw material for industrial processing for food and fuel. However, their analysis in a relevant laboratory can be costly, time-consuming, and not scalable. We, therefore, analyzed whether merging multispectral images and machine learning (ML) algorithms can develop a non-invasive, predictive framework to map canopy reflectance to °Brix and Purity. We acquired multispectral images data of a sugarcane-producing area *via* unmanned aerial vehicle (UAV) while determining °Brix and analytical Purity from juice in a routine laboratory. We then tested a suite of ML algorithms, namely multiple linear regression (MLR), random forest (RF), decision tree (DT), and support vector machine (SVM) for adequacy and complexity in predicting °Brix and Purity upon single spectral bands, vegetation indices (VIs), and growing degree days (GDD). We obtained evidence for biophysical functions accurately predicting °Brix and Purity. Those can bring at least 80% of adequacy to the modeling. Therefore, our study represents progress in assessing and monitoring sugarcane on an industrial scale. Our insights can offer stakeholders possibilities to develop prescriptive harvesting and resource-effective, high-performance manufacturing lines for by-products.

## 1 Introduction

Sugarcane (*Saccharum* spp.) is a semi-perennial grassy crop. Such a crop offers the global agriculture and bioeconomy sector possibilities to fabricate food, fuel, and feed (Barbosa Júnior et al., 2022a). It is a world-leading source of sugar for human consumption. In addition, it represents one of the most relevant renewable resources for bioenergy production, making it strategic for sustainable development (Yang et al., 2021). Full-scale fields across sugarcane-producing countries often yield 55 tons of stalk per hectare. Approximately 10–20% of its proximate composition is sucrose, while fiber contributes 10–15%, depending on technology and management (Hithamani et al., 2018; Sreedevi et al., 2018; Yang et al., 2019).

As sugarcane grows, it synthesizes and stores sugars throughout its structure to maintain physiological functions and overcome stresses (e.g., drought, salinity, and heat) under harsher conditions. however, it can significantly accumulate photo-assimilates only at maturity, which occurs between 10 and 18 months after planting, depending on genotype-environment interactions (Hithamani et al., 2018; Sreedevi et al., 2018; Yang et al., 2019). A mature plant can reflect the maximum incident solar radiation through the canopy, allowing its monitoring by a reflectance sensor. However, if it is over-mature or at flowering, its respiration increases. As a result, net photosynthesis and available sucrose in the stalk decrease, driving the need to determine an optimal time to harvest cost-effective material for industrial processing (Khan et al., 2022; Misra et al., 2022).

Chlorophylls are primary light-harvesting pigments. They can provide reliable indicators of the physiological conditions of a crop, such as sugarcane (Barbosa Júnior et al., 2022b). Therefore, evaluating them for fluorescence or measuring canopy reflectance can offer stakeholders possibilities to map and monitor the conversion of radiant energy to sucrose during ripening (Khan et al., 2022; Misra et al., 2022). In regular mechanical harvesting plans, staff estimates the degree of maturity by measuring °Brix and Purity. Such an intervention is effective; however, it can be costly, laborious, and time-consuming. In addition, it can be invasive, as it requires collecting stalks for juice extraction and technological analysis. An alternative to conventional sampling would be remote sensing. The technology can accurately and realistically capture spectral information without subjectiveness and destruction (Barbosa Júnior et al., 2022b).

By reviewing the literature on remotely sensing sugarcane, the system-level study by Bégué et al. (2010) can provide valuable information about the technical viability of forecasting sugarcane yield and sugar content upon imagery data. The authors integrated biometric measures and satellite time series into a framework. Then they tested its ability to model the spatio-temporal variability of those variables. Stages as late as maturation offered better phenological conditions to acquire multispectral images on the field than sprouting and tillering; hence, they allowed the most accurate forecasting of biomass yield and sugar content upon normalized difference vegetation index (NDVI). They developed other applicable predictors than NDVI, such as R, G, B, NIR, and SWIR. More importantly, they enhanced the performance of such single spectral bands and (VIs) by combining them with the leaf area index (LAI), supporting their hypothesis. However, their approach can require extensive radiometric inter-calibration to function. In addition, the remote sensing platform they employed to acquire data depends on the weather, driving the need to research a low-altitude crop-sensing device with a higher revisiting capacity.

Chea et al. (2020) analyzed whether an unmanned aerial vehicle (UAV) could acquire aerial remote sensing data to predict °Brix, Pol, and fiber. The authors mounted a multispectral sensor (R, G, B, NIR, and RedEdge) onboard equipment to develop a more detailed mission and calculate a suit of VIs, such as green normalized difference vegetation index (GNDVI), ratio vegetation index (RVI), chlorophyll index–green (CIG), chlorophyll index–rededge (CIRE), and simple ratio pigment index (SRPI), as alternatives to NDVI since it is sensitive to environmental noises (e.g., background brightness). Moreover, they added information about drought-tolerant and flood-tolerant genotypes to the biophysical modeling to improve the addressability of their approach. Models involving CIRE predicted °Brix and Pol most accurately (0.7< R^2^< 0.85). They could work better on processing data from a tolerant-drought field. However, they could not predict °Brix and Pol upon imagery data on a flood-tolerant area as accurately as those functions containing SRPI.

In a more recent publication, Chea et al. (2022) demonstrated the significance of machine learning (ML) algorithms to improve predicting °Brix on multiple-source data (i.e., agronomic, climatic, and spectral). The authors brought further information about the crop (i.e., size and age) and weather (i.e., precipitation) into the biophysical modeling to advance their research. Gradient boosting (GB) outperformed lasso, support vector machine (SVM), and random forest (RF) in describing °Brix upon spectral modifications in the canopy. It developed 70% accuracy and 3.3°Brix precision at processing only VIs, such as CIRE, green leaf index (GLI), and photosynthetic vigor ratio (PVR). However, combining these spectral predictors with agronomic and climate data could optimize its robustness (0.8< R^2^< 0.9; RMSE = 2.8°Brix). Therefore, UAV and ML could be enablers in soluble solids (SS) as indicators of maturity in sugarcane. However, Purity could offer a more reliable marker than °Brix in mapping and monitoring saccharification. It describes the proportion of sucrose the juice contains and is an indicator of raw material degradation during the cut-to-crush time and industrial processing efficiency.

Therefore, we analyzed whether ML algorithms could predict °Brix and Purity upon multispectral UAV imagery data for precision mechanical harvesting of material with higher quality.

## 2 Material and methods

### 2.1 Site description and field data collection

**Figure 1:**
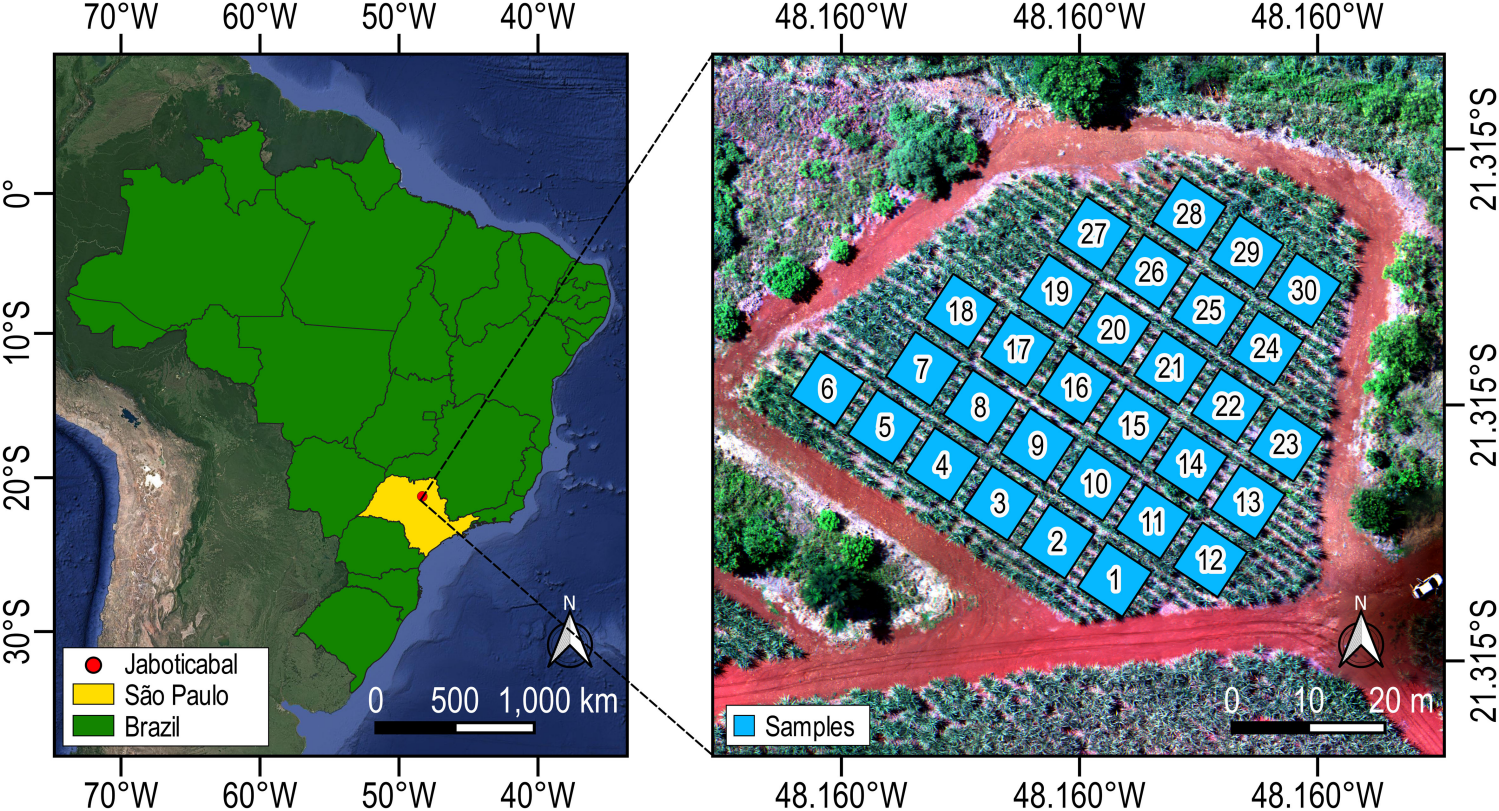
Brazil map highlighting the study region (left). UAV orthomosaic of the study field with sample plots for field and spectral data collection (right).

We carried out our study in a sugarcane field located near the city of Jaboticabal, São Paulo, Brazil (**Figure 1**). The region has an Oxisol type soil with low slope (0 - 8%). The climate of the region is of type Aw with a summer dry season. Annually, rainfall reaches about 1460 mm and the average temperature is 22.6°C. We conducted our study with the cultivar RB 97-5201 in sixth ratoon. We performed 8 samplings throughout the maturity stage of the crop (beginning February 28 and ending May 8, 2022) with an interval between samplings of 15 days. In each analysis, data were collected at 30 sample points regularly distributed (9 x 9 m grid) and spaced 2 m apart (**Figure 1**). On evaluation days we captured images with UAV and randomly collected 4 stalks at each sample point. In total, our dataset was composed of 240 samples (30 samples x 8 dates). The images were processed and the stalks were sent to the laboratory for analysis of °Brix and Purity contents. Additionally, we included growing degree days (GDD) information to establish functional relationships with crop phenology.

### 2.2 Flight campaign and spectral features collection

A multirotor UAV (DJI Phantom 4 Multispectral RTK, Shenzhen, China) was used as the remote sensing platform in this study. The UAV is equipped with a multispectral camera that has five spectral bands, namely Blue (450 nm ± 16 nm), Green (560 nm ± 16 nm), Red (650 nm ± 16 nm), RedEdge (730 nm ± 16 nm), and NIR (840 nm ± 26 nm). The UAV has a sunlight sensor on top to compensate for incident solar radiation during flight and ensure that spectral data are consistent. In addition, it is equipped with a multi-frequency GNSS receiver (DJI D-RTK2 base Station, Shenzhen, China) able to receiver signals from constellation namely GPS, GLONASS, BeiDou, and Galileo, ensuring centimeter positional accuracy, making it possible to acquire temporal data from the same point. The flight missions were performed automatically by application (DJI GS Pro, Shenzhen, China). Flight settings and parameters are described in **Table 1**.

**Table 1:** Flight guideline and specifications.

Date	Time		Ground Sample Distance - GSD (cm)
	Start	End	
02/28/2022	02:25 PM	02:29 PM	3.14
03/15/2022	02:07 PM	02:11 PM	3.15
03/29/2022	01:53 PM	01:57 PM	3.16
04/12/2022	02:23 PM	02:27 PM	3.21
04/25/2022	11:29 AM	11:33 AM	3.22
05/10/2022	02:10 PM	02:14 PM	3.18
05/24/2022	11:26 AM	11:30 AM	3.20
06/08/2022	02:10 PM	02:14 PM	3.20

Flight height: 60 m, images overlap: 75%, speed: 5.2 m/s, and number of images: 500.

The images were stitched using Structure from Motion (SfM) software (Agisoft Metashape Professional 1.5.5, Agisoft, St. Petersburg, Russian) to generate 8 multispectral orthomosaics. To extract the spectral information and calculate the vegetation indices (**Table 2**) we used the open-source package “FIELDimageR” (Matias et al., 2020); in the programming language R (version 4.1.0).

**Table 2:** Vegetation indices used in this study.

Index	Nomenclature	Equation	Reference
BGI	Blue Green Pigment Index	$\frac{Blue}{Green}$	(Zarco-Tejada et al., 2005)
CIRE	Chlorophyll Index – RedEdge	$\frac{NIR}{RedEdge}$	(Gitelson et al., 2003)
CIVE	Color Index of Vegetation Extraction	0.441·*Red* – 0.811·*Green* +0.385·*Blue* + 18.79	(Kataoka et al., 2003)
GLI	Green Leaf Index	$\frac{2\cdot Green-Red-Blue}{2\cdot Green+Red-Blue}$	(Louhaichi et al., 2001)
GNDVI	Green Normalized Difference Vegetation Index	$\frac{NIR-Green}{NIR+Green}$	(Gitelson et al., 1996)
NDVI	Normalized Difference Vegetation Index	$\frac{NIR-Red}{NIR+Red}$	(Rouse et al., 1974)
PSRI	Plant Senescence Reflectance Index	$\frac{Red-Green}{RedEdge}$	(Merzlyak et al., 1999)
TVI	Triangular Vegetation Index	0.5·(120·(*NIR* – *Green*) –200·(*Red* – *Green*))	(Broge and Leblanc, 2001)
VARI	Visible Atmospherically Resistant Index	$\frac{Green-Red}{Green+Red}$	(Gitelson et al., 2002)

### 2.3 Laboratory analysis

After the collection of stalks in the field, they were properly identified and taken to the laboratory to determine the quality parameters °Brix and Purity. Initially, the stalks from each sample point were processed individually in a hydraulic press for juice extraction. We used the juice to measure the °Brix content by digital refractometer (ABBE, Atago Pal-1, Tokyo, Japan) and recorded the value corrected to a temperature of 20°C. To measure Purity, we followed the methodology proposed by Consecana (2006). We diluted 10g of clarifying substance based on aluminum chloride in 200 mL of juice. The solution was filtered and the measured value was recorded using a polarimeter (Anton Paar, Bremen, Germany). To determine purity we used Equations 1, 2, and 3.


(1) LPb=1.0078·LAI+0.0444

(2) %Pol=LPb·(0.2605−0.0009882·Brix)

(3) $$Purity\;\left(\%\right)=\frac{\%Pol}{Brix}\cdot 100$$

Where, *LPb* is the polarimetric reading equivalent to lead subacetate, *LAI* is the polarimetric reading with aluminum chloride, and *%Pol* is the apparent sucrose content.

### 2.4 Data analysis

#### 2.4.1 Data curation

A total of 15 independent variables (including GDD, five spectral bands and nine VIs) were used as input to the °Brix and Purity prediction models. For the data to faithfully represent the field truth, we applied the interquartile range method to remove outliers from the dataset. Thus, the length of our dataset was reduced from 240 to 223. Then the dataset was randomly divided into subsets with 70% (156) and 30% (67) for train and test, respectively. Since we constructed our dataset with 15 predictor variables for °Brix and Purity, we decided to apply the *best subsets regression* function from the open-source package “olsrr” (Neter et al., 1996), in the programming language R (version 4.1.0), to select the best features for predicting °Brix and Purity. The *best subsets regression* is a selection approach that consists of testing all possible combinations of the predictor variables and then selecting the best among them to constitute a future model. This technique can effectively select the independent variables that contribute significantly to the change in a dependent variable. The features selection was done based on the coefficient of determination (R²) and mean squared error of prediction (MSEP).

#### 2.4.2 Machine learning algorithms

To model the contents of °Brix and Purity we chose 4 ML regression algorithms, namely multiple linear regression (MLR), random forest (RF), decision tree (DT) and support vector machine (SVM). These algorithms are widely used because they produce high accuracy results, solve problems on relatively small database sizes and handle a large number of input features. All analyses were performed in the programming language R (version 4.1.0) using the packages “stats” (Wilkinson and Rogers, 1973), “randomForest” (Breiman, 2001), “rpart” (Breiman et al., 1984); and “e1071” (Rong-En et al., 2005); for the algorithms described above, respectively. Hyperparameters are described in **Supplementary Table 1**.

#### 2.4.3 Model evaluation and validation

The fit of the models was evaluated according to the coefficient of determination (R²), root mean square error (RMSE) and mean absolute error (MAE) applied to the test dataset. The closer the R² value is to 1, the more precise. In contrast, the closer the RMSE and MAE values are to 0, the more accurate the model.

## 3 Results

### 3.1 Spatio-temporal evolution of °Brix and Purity

**Figure 2:**
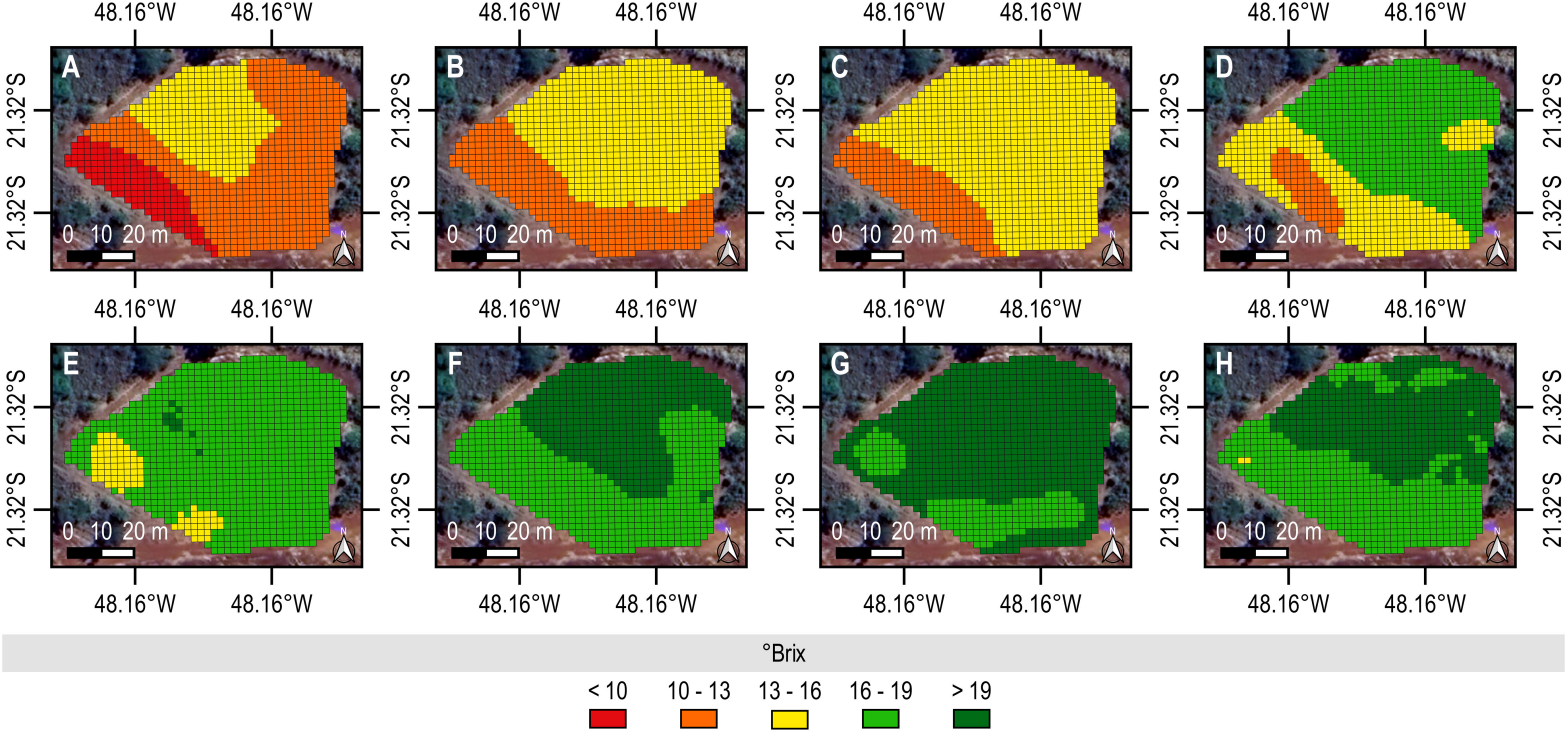
Spatio-temporal mapping of °Brix from ground-level biometric data. The values upscale as the color changes from scarlet to emerald. In addition, sublevel charts provide digital representations of sampling dates. **(A)** was the first data collection and **(H)** was the last data collection. The reference data set was used to construct the maps by the ordinary kriging interpolation method (2 x 2 m) performed in the QGIS (version 3.22.5) using the “Smart-Map” plugin (Pereira et al., 2022).

**Figure 3:**
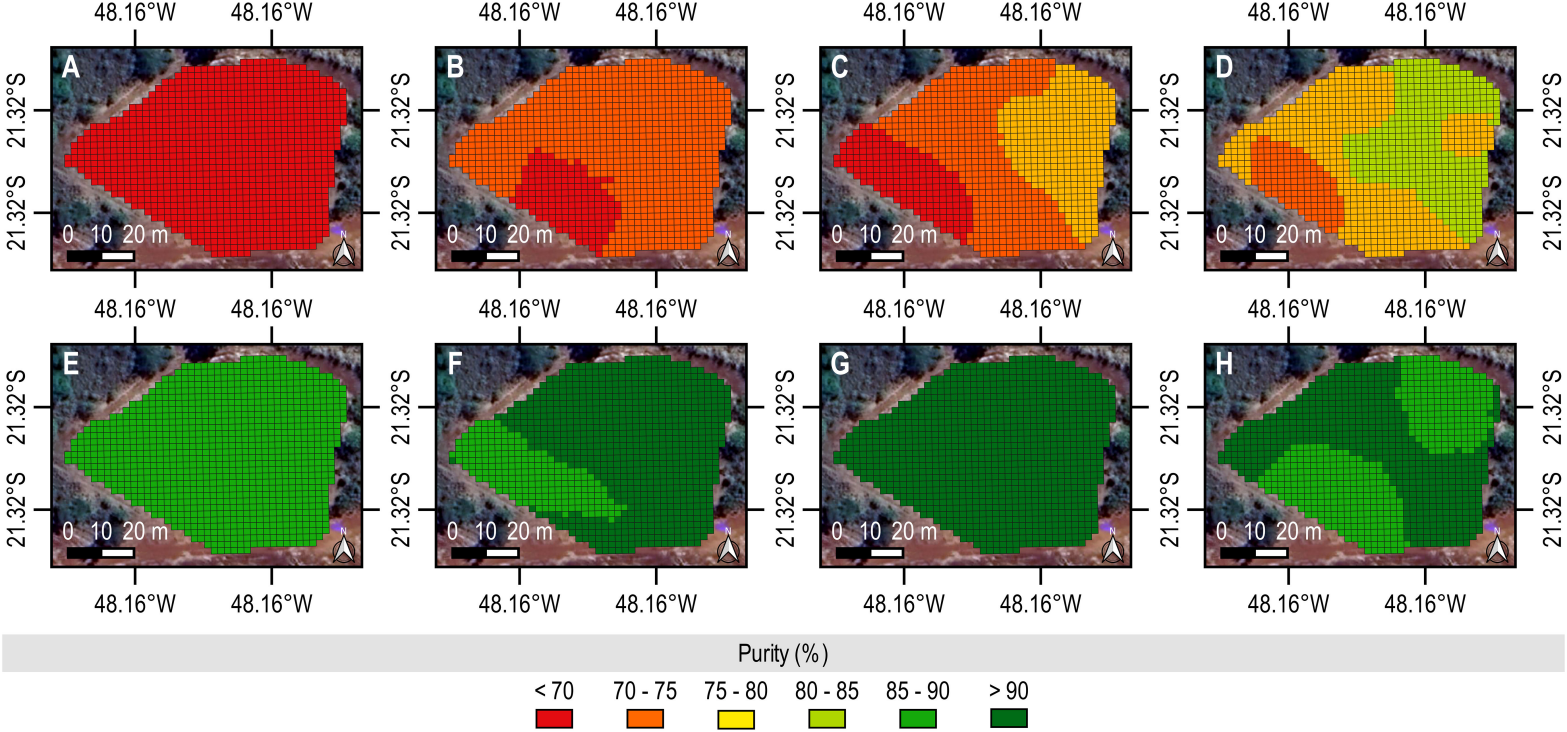
Spatio-temporal mapping of Purity from ground-level biometric data. The values upscale as the color changes from scarlet to emerald. In addition, sublevel charts provide digital representations of sampling dates. **(A)** was the first data collection and **(H)** was the last data collection. The reference data set was used to construct the maps by the ordinary kriging interpolation method (2 x 2 m) performed in the QGIS (version 3.22.5) using the “Smart-Map” plugin (Pereira et al., 2022).

We mapped the dynamic ripening on biometric data (**Figures 2** and **3**). As the crop ripened, it accumulated SS in the stalk; hence, the °Brix (**Figure 2**) and Purity (**Figure 3**) of analytical juice increased temporally and spatially. For instance, °Brix initially was 12.8 ± 2.5. Such a measure of SS then rose to 14 ± 1.7 at the 2^nd^ evaluation. Additionally, we measured 14.4 ± 2.1°Brix from samples of the 3^rd^ collection, supporting a field at early maturity and still unsuitable for cost-effective harvesting. As the elongation occurred, however, the °Brix increased significantly. Therefore, its values for the 4^th^ and 5^th^ survey-level evaluations were 16.9 ± 2.1 and 17.9 ± 1.6, respectively. Summarily, sugarcane developed the highest °Brix of 19.7 ± 0.9 at the 7^th^ evaluation.

We identified a similar trend to Purity. The crop produced 80–85% pure juice until the 4^th^ evaluation. Later, in the 7^th^ evaluation, however, the measure for this technological feature exceeded 90%, supporting an optimal Pol/SS ratio for high-quality harvesting. More importantly, its distribution throughout the field was homogeneous, further supporting the suitability of such a phenological stage for standard operation and precision crop management. A decreasing proportion of the area with the highest °Brix and Purity at the 8^th^ evaluation could make the recovery of adequate raw material (sucrose) for industrial processing difficult, driving the need to determine the most reliable time to intervene in the field. Therefore, by analyzing the spatio-temporal variability of such indicators of qualitative yield, we must plan to harvest the sugarcane at the 7^th^ evaluation. However, we could act earlier since 50–70% of the area produced a raw material with 18–19°Brix and 85–90% purity at the 6^th^ evaluation.

### 3.2 Selecting spectral predictors of °Brix and Purity

**Figure 4:**
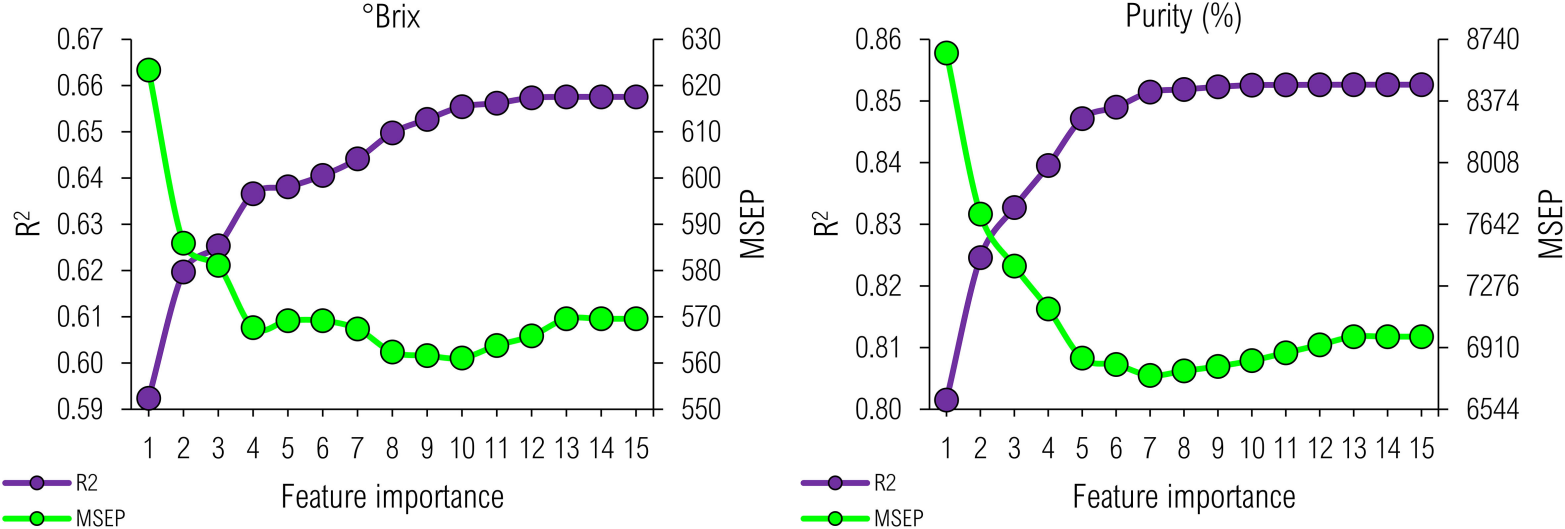
Best Subsets Regression performance to predict °Brix and Purity (%).

We selected spectral features to predict °Brix and Purity by applying regression analysis to remote sensing data (**Figure 4**). A spectral band or VI capable of predicting °Brix could not provide an accurate predictor of Purity and vice versa, supporting structural input-to-output dependencies and particularities of such indicators of qualitative yield. For instance, Blue, Red, and NIR contributed to developing an adequate ten-input predictive model for °Brix. However, they could not function as accurately as Green and PSRI in predicting Purity through a topologically less complex function consisting of seven predictors (**Table 3**). Such a single band and VI contributed to bringing an R^2^ of 0.85 into the biophysical modeling for Purity, while the adequacy for those above at predicting °Brix was 0.65, making them less accurate.

**Table 3:** Best predictors of °Brix and Purity upon imagery data.

Parameters of quality yield	Predictors	R^2^	MSEP
Brix	Blue Red RedEdge NIR CIRE GNDVI NDVI GLI VARI GDD	0.65	561.09
Purity	Green RedEdge CIRE GNDVI PSRI VARI GDD	0.85	6744.96

By analyzing MSEP, however, we could recognize a lower predictive error from such a brix-fitting model, making it more precise. In addition, the more inputs, the higher the accuracy and precision of a polynomial function (**Figure 4**); however, its complexity can increase, potentially forcing an ML algorithm to misfit data through either underfitting or overfitting a trend. A higher number of inputs usually implies a higher degree of freedom; hence, a model becomes more robust and probable to reject a false hypothesis and produce significant output. However, further increasing the number of predictors could not increase precision (**Supplementary** **Table 2** and **3**), supporting the occurrence of multicollinearity or correlation between them (**Supplementary Figure 1**). Mutual relationships commonly reduce predictive performance in statistical modeling, driving the need to re-design or exclude part of them (Lindner et al., 2022). However, if stakeholders understand the role of independent variables, constraining them in an ML model to reduce multicollinearity is unnecessary. In such a case, it can neither determine exactness and generalization nor result in misinterpretation and misinformation (Lindner et al., 2022). Therefore, balancing adequacy and complexity is significant in addressing the biophysical modeling of °Brix and Purity upon imagery data without computational unfeasibility.

### 3.3 Performance of machine learning models at predicting °Brix and Purity upon imagery data

**Figure 5:**
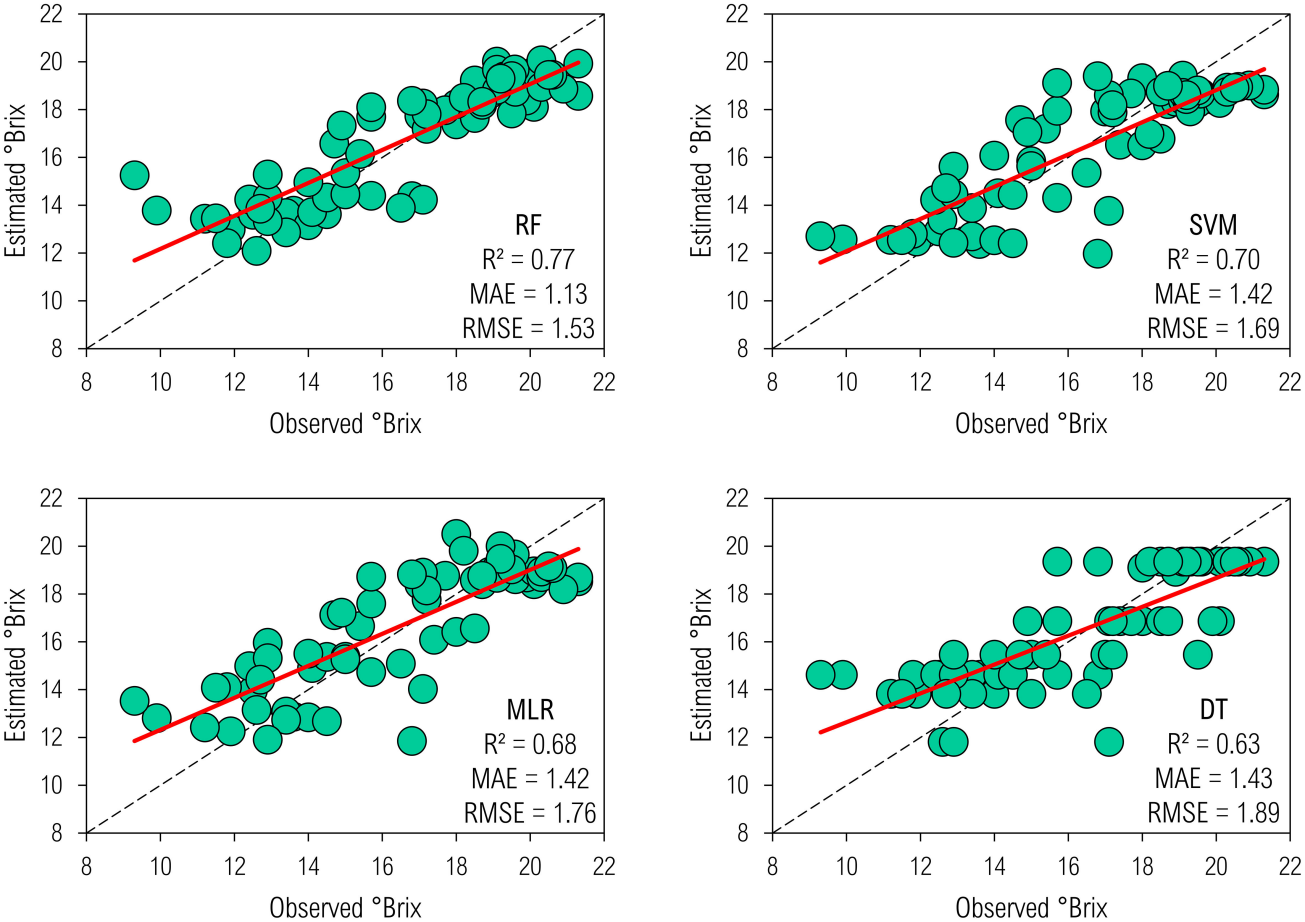
Biophysical modeling of °Brix by machine-learning regressors.

**Figure 6:**
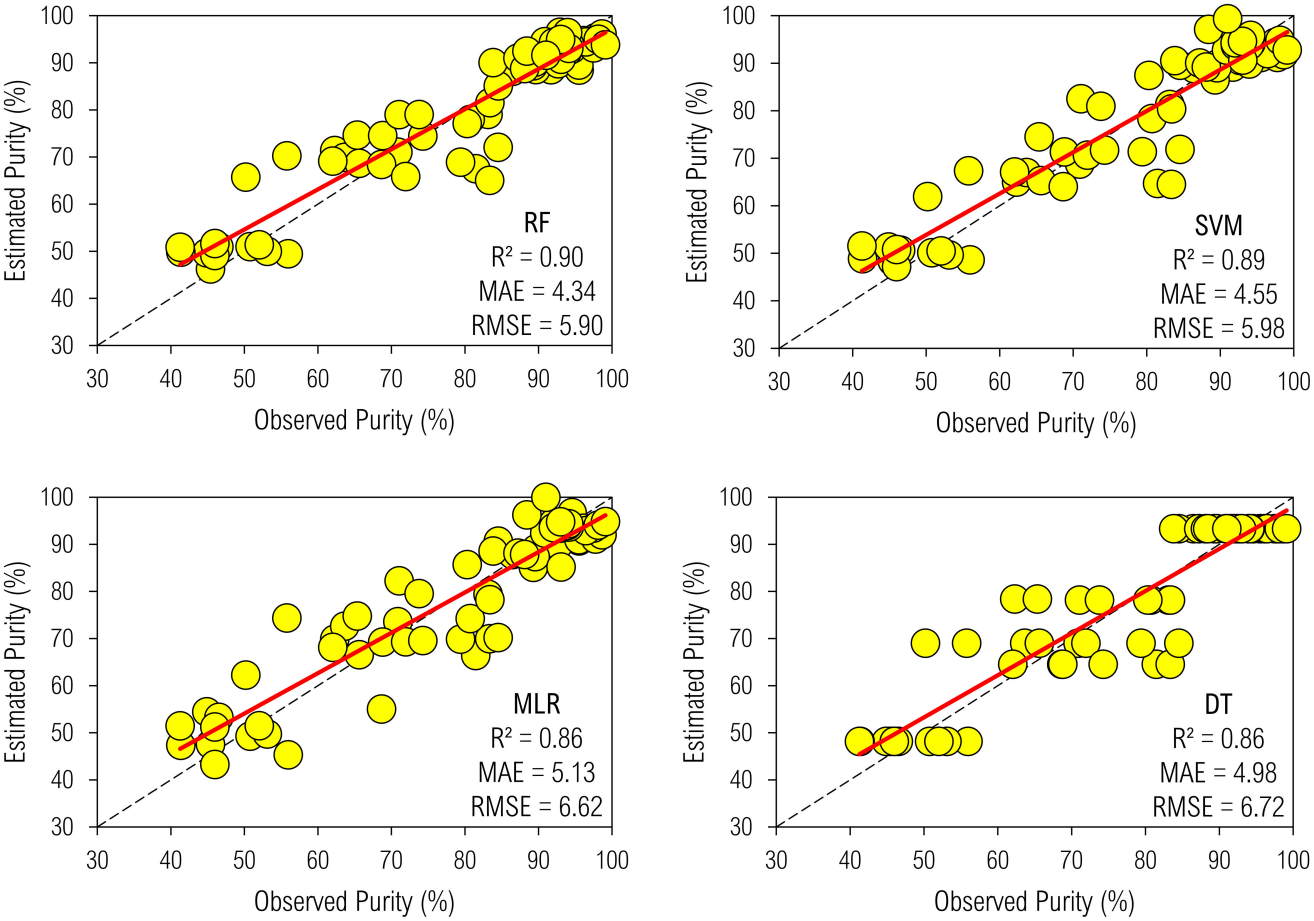
Biophysical modeling of Purity by machine-learning regressors.

Machine-learning models effectively estimated °Brix (**Figure 5**) and Purity (**Figure 6**) by processing biometric and remote sensing data. They were as precise as accurate, allowing the selection of a non-linear function to best describe qualitative yield, logically, irrespective of the indicator. Random forest brought the highest R^2^ into the biophysical modeling for °Brix; hence, it qualified as the most accurate algorithm. In addition, SVM estimated such a measure of SS as accurately and precisely as RF, outperforming both MLR and DT. These approaches developed the least accuracy and precision as ten-input regressors.

We obtained mathematical descriptions with higher precision for Purity since estimates of R², MAE, and RMSE were 0.85–0.9, 4.3–5.1%, and 5.9–6.7%, respectively; the ranges of these metrics for ML models predicting °Brix were 0.6–0.8, 1.1–1.4%, and 1.5–1.9%, respectively. Therefore, compared to Purity, such an indicator of the technological quality of juice added more systematic errors in the modeling, reducing exactness; however, it could not necessarily decrease correctness, which is another part of robustness. Random forest most accurately and precisely predicted not only °Brix but also Purity, further supporting its outperformance at learning on spatio-temporal data to map a series of spectral inputs to an agronomic output. Its predictive metrics were 0.9 R², 4.6% MAE, and 6% RMSE. Additionally, MLR and DT described the Purity at an equal level of adequacy. However, DT outperformed the MLR in supporting a seven-input model to predict Purity.

## 4 Discussion

### 4.1 Spatio-temporal evolution of °Brix and Purity and its implications to precision harvesting

As sugarcane ripens, it accumulates sugars in organs throughout its structure, such as leaves, stalks, and roots. However, as it grows and develops vegetatively and reproductively, it significantly consumes them to sustain its physiological functions (Khan et al., 2022). In advanced phenological stages, it transports photoassimilates from older (or senescent) leaves to younger parts, such as the stalk in its parenchymal cells and vacuoles (Misra et al., 2022). While these compartments act as sucrose reservoirs in a mature plant, the flowering can manifest as a sink in an over-mature plant, decreasing its content. We could reproduce and visualize these dynamics on maps of °Brix (**Figure 2**) and Purity (**Figure 3**). We quantified the highest quantity of SS from samples of the 7^th^ evaluation; hence, they produced the purest juice. However, °Brix and Purity decreased as the field started flowering at the 8^th^ evaluation. In addition, stalks became more fibrous, supporting “isoporization” (Morais et al., 2015). Such a phenomenon indicates a reduction in water and sugar, making harvesting inefficient and costly (Poltroniere et al., 2021). By monitoring the field and mapping °Brix and Purity, we can offer stakeholders possibilities to optimize their on-farm management and agribusiness models.

Stakeholders usually rely on sugary substrates to recover sugarcane from the field cost-effectively. °Brix provides a reliable measure of SS in material, while Purity indicates the portion of sugar it contains. Therefore, both are significant technological features for farmers and sugar-energy plants to decide on activities and processes. Raw material with high °Brix and Purity is desirable for commercialization. However, if it excessively consists of minerals and sugars other than sucrose, such as glucose and fructose, its Purity becomes lower than usual, making harvesting and industrial processing challenging. Relevant standardization bodies of the sugar-energy sector in Brazil and abroad set °Brix and Purity to be higher than 18 and 85%, respectively, for economically sustainable mechanical harvesting. Sugarcane can develop such optimal values before or at physiological maturity. However, as it is a semi-perennial grass, it continues to vegetate during ripening; hence, these indicators of qualitative yield decrease nonlinearly over time and spatially, as evidenced by our prescriptive maps (**Figures 2** and **3**). Prospective producers who search for precision farming support systems can ground their analytical (not empirical) decisions and actions in these digital representations of an experimental field. Perhaps, they harvest material for making food and fuel with higher accuracy and better quality while optimizing workflow.

### 4.2 Relationships between spectral features and indicators of qualitative yield

Spectral features offer stakeholders reliable markers to monitor and map crops. They respond to modifications in nutritional composition (Shendryk et al., 2020), accumulation of biomass (Abebe et al., 2022), and physiological events of maturation (Chea et al., 2020). Single bands and their mathematical combinations into VIs allow for collecting significant imagery data on agroecosystems, whether to make decisions on operations from implementation (e.g., seeding and planting) to harvesting. Researchers often exploit them in remotely assessing the agronomic performance of sugarcane for biomass (Wang et al., 2022), quantitative yield (Sumesh et al., 2021), and standard biometric variables, such as leaf area and height of an individual (Oliveira et al., 2022). However, they still have not emphasized applying ML to UAV imagery data to predict °Brix and Purity as we focus on. Therefore, our AI-intensive approach is innovative. It can realistically monitor saccharification on canopy reflectance during ripening, as photosynthetically active leaves determine stalk sugar concentration (Khan et al., 2022). In addition, it can offer accurate and precise biophysical models to establish functional relationships between spectral features and indicators of technological quality (**Table 3**).

We obtained evidence for Red and NIR improving the robustness of brix-predicting models. Wavelengths occurring in the electromagnetic radiation spectrum around 680 nm and above visible red light between 780 nm and 1 mm can manifest as exciters to chlorophylls, inducing them to emit either photon (reflectance) in a specific spectral band or fluorescence within a region (Jensen, 2009; Zhao et al., 2010; Stein et al., 2014; Silva Junior et al., 2018; Rodrigues et al., 2020; Barbosa Júnior et al., 2022b). Moreover, they can correlate with the concentration of nutrients (e.g., sugars and minerals) in parts of a plant, such as a stalk (Rodrigues et al., 2022), supporting the ability of our models to predict °Brix and Purity upon imagery data. However, Red and NIR could not estimate Purity as accurately and precisely as Green and RedEdge. As sugarcane grows, its photosynthetic activity intensifies, triggering chemical modifications to chloroplasts. The accumulation of sugars from leaves in the stalk further contributes to physiological reactions in these membrane-bound organelles, altering the balance of chlorophylls and the “greenness” of a plant (Chea et al., 2022). hence, we can acquire significant spectral data from a canopy to predict Purity, which provides a measure of available sucrose in SS.

### 4.3 Machine learning models for predicting °Brix and Purity upon imagery data

Predictive data analytics can develop knowledge for advancing agriculture. However, conventional models can be statistically complex and demand considerable computational processing, making their implementation challenging. Even though fundamental approaches, such as correlational or regression analysis (Chea et al., 2020; Todd et al., 2022), can determine functional relationships between spectral and agronomic features, they could not be mathematically sufficient to address problems with a high level of abstraction. Therefore, their application in complex farming systems could not be cost-effective, driving the need to develop an alternative to explain non-linear interactions.

We can train an ML algorithm on a heterogenous and “messy” dataset to learn meaningful and non-duplicative patterns to solve a task automatically, accurately, and unbiasedly. Some applications of ML for sugarcane research and development available from earlier independent studies include predicting or forecasting chlorophyll content (Narmilan et al., 2022), standard morphophysiological variables (Oliveira et al., 2022), production of biomass (Wang et al., 2022), and classify cultivation (Nihar et al., 2022). We developed a new pathway by mapping spectral features to °Brix and Purity; hence we can fulfill a gap in analyzing qualitative yield while improving the addressability of a UAV for scalable aerial remote sensing. Our models are accurate and precise, especially RF and SVM. RF performs an independent prediction by processing data through multiple decision trees (Breiman, 2001). Support vector machine maps inputs to output as a classifier rather than as a regressor (Cristianini and Shawe-Taylor, 2000). As RF provides more parameters and higher overfitting prevention capability for ML, it can outperform SVM in predictive analysis (Yuan et al., 2022), supporting our trends.

Decision tree and MLR could be options for RF and SVM in predicting °Brix and Purity. However, they could develop a lower level of accuracy or precision, driving the need for improvement. The DT consists of an advanced problem-solving and computation-performing procedure. It splits a dataset into multiple branches to establish relationships hierarchically (Ghosh et al., 2022). However, such an algorithm has an inherent flaw, causing it to be less effective. Therefore, implementing a flawless filter could be necessary to increase its accuracy and precision in processing data with significant fluctuations. Even though MLR is topologically and operationally more basic than other ML algorithms, it can develop a highly accurate predictive model for Purity. Such a technique can work well on linear imagery data; hence, it can offer a reliable estimate of quantitative variables, such as productivity, upon VIs (Todd and Johnson, 2021; Krupavathi et al., 2022). However, it could not predict °Brix as accurately as RF and SVM, supporting a non-linear dataset. By introducing GDD into the model, however, we can optimize its predictive performance. Sugarcane’s GDD varies proportionally to its growth and development, acting as a source of constant propagation to MLR.

### 4.4 Advantages, trade-offs, and implications

We demonstrated the technical viability of ML algorithms in predicting °Brix and Purity upon UAV imagery data. Our approach is still at an early stage of research and development. However, it is consistent and can offer stakeholders possibilities to address precision harvesting for cost-effective production. Such an operation is costly (Banchi et al., 2019) and determines the quantity and quality of material for industrial processing (Martins et al., 2021). Therefore, prospective stakeholders across researcher centers and industries who search for decision-making support systems can benefit from our AI-intensive biophysical models to predict the optimal time for harvesting. As sugarcane fulfills approximately 80% of global sugar production (FAOSTAT, 2020), recovering material with the highest quality possible from the field at the precise time and place can be significant to develop a thriving and responsive sugar-energy sector.

Acquiring imagery data by a multispectral sensor onboard UAV allows the development of accurate and precise biophysical modeling of qualitative yield. Our predictive frameworks can be technically comparable with those functions available in independent studies by Bégué et al. (2010), Chea et al. (2020); and Chea et al. (2022). However, they can offer farmers further information to monitor dynamic ripening and map regions of high °Brix and Purity for “smart” harvesting. In addition, our approach can work by processing only remote sensing data, not depending on a conventional ground-level survey to collect biometric measures. Therefore, such an advantage can save farmland staff time and labor, streamline workflow, and ultimately level up the cost-effectiveness of production. Furthermore, while our approach can predict qualitative yield, it can be part of a high-throughput phenotyping program to select early-maturity genotypes. Stakeholders often rely on passive sensors to monitor and assess breeding fields, opening the opportunity to investigate active devices for this purpose.

## 5 Conclusion

We predicted °Brix and Purity by applying machine learning to multispectral imagery data from a UAV. We optimized the biophysical modeling by implementing a random forest algorithm. The most accurate spectral predictors of °Brix were Red and NIR, while those of Purity Green and RedEdge. We, therefore, developed an AI-intensive solution to model qualitative yield, advancing the field of aerial remote sugarcane mapping and monitoring. Our approach offers the global sugar-energy sector a strategy to harvest high-quality feedstock for industrial processing while streamlining fieldwork and addressing a pressing prescriptive and analytical agriculture for sustainable development. Additionally, it provides knowledge to develop a resource-effective, self-evolving framework to select sugar-dense material objectively and non-invasively, which is not an assumption of conventional phenotyping.

## Data availability statement

The raw data supporting the conclusions of this article will be made available by the authors, without undue reservation.

## Author contributions

MBJ: conceptualization, methodology, validation, formal analysis, investigation, data curation, writing – original draft preparation, writing – review and editing, and visualization. BM: methodology, formal analysis, investigation, writing – original draft preparation, writing – review and editing, and visualization. RO: investigation, writing – review and editing, and visualization. LS: writing – review and editing, visualization, and supervision. RS: conceptualization, methodology, writing – review and editing, visualization, supervision, and project administration. All authors contributed to the article and approved the submitted version.
