# Supplementary material for: UAV imagery data and machine learning: A driving merger for predictive analysis of qualitative yield in sugarcane
Source: Front Plant Sci. 2023 Jan 26;14:1114852. doi: 10.3389/fpls.2023.1114852 (PMC9929953; doi:10.3389/fpls.2023.1114852)
Supplement: Supplementary file 1 [file DataSheet_1.pdf]

Supplementary Material

Supplementary Table 1. Hyperparameter tuning of machine learning algorithms.

Acronym	ML Algorithm	Hyperparameter Usage
MLR	Multiple Linear Regression	lm(formula, data)
RF	Random Forest	randomForest(formula, data, mtry = 7.44, ntree = 1000)
DT	Decision Tree	rpart(formula, data)
SVM	Support Vector Machine	svm(formula, data, type = "nu-regression", kernel = "linear")

Supplementary Table 2. Best Subsets Regression performance to predict °Brix.

n	predictors	R^2	MSEP
1	GDD	0.59	623.36
2	RedEdge GDD	0.61	585.93
3	CIRE TVI GDD	0.62	581.13
4	Red RedEdge TVI GDD	0.63	567.65
5	Red RedEdge NIR GLI GDD	0.63	569.16
6	Red RedEdge NIR CIRE GLI GDD	0.64	569.15
7	Blue Red CIRE GNDVI NDVI GLI GDD	0.64	567.38
8	Blue RedEdge NIR GNDVI NDVI TVI GLI GDD	0.64	562.38
9	Blue RedEdge NIR GNDVI NDVI TVI GLI VARI GDD	0.65	561.60
**10**	**Blue Red RedEdge NIR CIRE GNDVI NDVI GLI VARI GDD**	**0.65**	**561.09**
11	Blue Red RedEdge NIR CIRE GNDVI NDVI PSRI GLI VARI GDD	0.65	563.80
12	Blue Red RedEdge NIR CIRE GNDVI NDVI PSRI BGI GLI VARI GDD	0.65	565.87
13	Green Red RedEdge CIRE GNDVI NDVI PSRI TVI BGI CIVE GLI VARI GDD	0.65	569.57
14	Green Red RedEdge NIR CIRE GNDVI NDVI PSRI TVI BGI CIVE GLI VARI GDD	0.65	569.57
15	Blue Green Red RedEdge NIR CIRE GNDVI NDVI PSRI TVI BGI CIVE GLI VARI GDD	0.65	569.57

Supplementary Table 3. Best Subsets Regression performance to predict Purity (%).

n	predictors	R^2	MSEP
1	GDD	0.80	8659.22
2	Green GDD	0.82	7702.34
3	Blue Green GDD	0.83	7394.26
4	Green NIR GNDVI GDD	0.84	7139.84
5	Green NIR GNDVI CIVE GDD	0.85	6847.67
6	Green RedEdge CIRE GNDVI CIVE GDD	0.85	6809.74
**7**	**Green RedEdge CIRE GNDVI PSRI VARI GDD**	**0.85**	**6744.96**
8	Green RedEdge CIRE GNDVI PSRI GLI VARI GDD	0.85	6771.77
9	Red RedEdge CIRE GNDVI PSRI BGI CIVE VARI GDD	0.85	6798.61
10	Red RedEdge CIRE GNDVI PSRI TVI BGI CIVE VARI GDD	0.85	6833.06
11	Blue Green RedEdge CIRE GNDVI PSRI TVI BGI CIVE VARI GDD	0.85	6879.04
12	Blue Green RedEdge CIRE GNDVI NDVI PSRI TVI BGI CIVE VARI GDD	0.85	6926.37
13	Blue Green RedEdge CIRE GNDVI NDVI PSRI TVI BGI CIVE GLI VARI GDD	0.85	6975.07
14	Blue Green Red RedEdge NIR CIRE GNDVI NDVI PSRI TVI BGI GLI VARI GDD	0.85	6975.07
15	Blue Green Red RedEdge NIR CIRE GNDVI NDVI PSRI TVI BGI CIVE GLI VARI GDD	0.85	6975.07

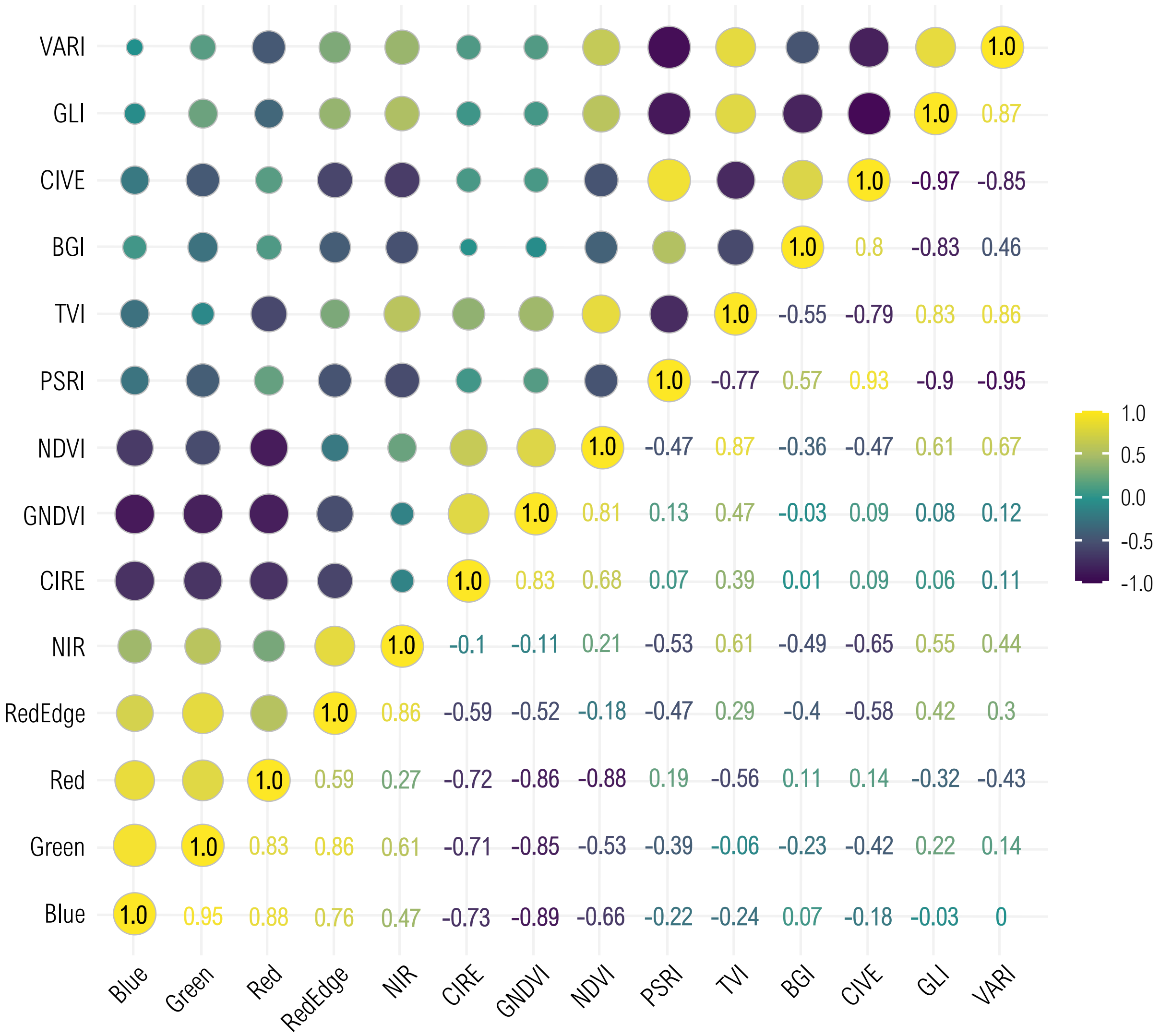

Supplementary Figure 1. Correlation between the input spectral features.
